# Employment and family caregiving in palliative care: An international qualitative study

**DOI:** 10.1177/02692163221089134

**Published:** 2022-06-06

**Authors:** Clare Gardiner, Beth Taylor, Hetty Goodwin, Jackie Robinson, Merryn Gott

**Affiliations:** 1 Health Sciences School, The University of Sheffield, Sheffield, UK; 2 School of Nursing, The University of Auckland, Auckland, Aotearoa New Zealand

**Keywords:** Palliative care, end of life care, family caregivers, informal caregivers, carers, employment

## Abstract

**Background:**

Family caregivers provide the majority of palliative care. The impact of family caregiving on employment and finances has received little research attention in the field of palliative care.

**Aim:**

The aim of this study was to explore perspectives and experiences of combining paid employment with palliative care family caregiving, and to assess the availability and suitability of employment support across three countries – the United Kingdom (UK), Aotearoa New Zealand and Canada.

**Design:**

A qualitative descriptive study design was used. Semi-structured interviews were held with 30 key informants with professional or personal experience in palliative care from the UK (*n* = 15), Aotearoa New Zealand (*n* = 6) and Canada (*n* = 9). Interviews were recorded, transcribed and analysed using the principles of thematic analysis.

**Results:**

Four main themes were identified: (1) significant changes to working practices are required to enable end of life family carers to remain in work; (2) the negative consequences of combining caregiving and employment are significant, for both patient and carer; (3) employer support for working end of life caregivers is crucial but variable and; (4) national, federal and government benefits for working end of life family carers are necessary.

**Conclusion:**

Supporting carers to retain employment whilst providing care has potential benefits for the patient at end of life, the caregiver, and the wider economy and labour market. Employers, policymakers and governments have a role to play in developing and implementing policies to support working carers to remain in employment.

**What is already known about the topic?**

- Family caregivers provide the majority of palliative care.
- Palliative care family caregiving has a significant impact on a family caregivers physical and psychological health.
- The impact of family caregiving on employment and finances has received little research attention in the field of palliative care.

**What this paper adds?**

- The study demonstrated that family caregiving in palliative care has a significant impact on carers employment.
- Changes often need to be made to accommodate caregiving alongside paid work, which has negative consequences for both patient and carer.
- Support from both employers and government is important for helping family carers remain in employment.

**Implications for practice, theory or policy**

- Supporting caregivers to remain in paid employment has potential benefits for the patient with palliative care needs, the caregiver, and the wider economy and labour market.
- Employers, policymakers and governments have a role to play in developing and implementing policies to support working carers to remain in employment.
- There is considerable potential for policymakers to learn from the experiences of other counties, by adapting existing models for caregiver and employment support.

## Introduction

Whilst palliative care is provided by a range of health and social care professionals family caregivers, who may or may not be related to the patient, provide the majority of this care. Evidence suggests family caregivers provide 75%–90% of care for people who have palliative care needs, both for those being cared for in hospital and in their own homes.^1^ In the UK, family carers of people in the last 3 months of life have been found to spend up to 70 h a week on informal caregiving tasks including healthcare, emotional and social support, and household tasks.^2^ Evidence from Canada suggests that during the last 4 months of life, family caregivers spend around 5829 h on unpaid caregiving.^3^

Demand for family caregiving is increasing due to rapidly ageing populations.^4^ In addition, recent shifts in palliative care policy across high income countries have seen a focus on re-locating palliative care provision out of hospitals and into communities.^5^ Undoubtedly, enabling patients to remain at home at the end of their lives has many benefits, yet the implications for family caregivers are profound, as the vast majority of home-based palliative care is undertaken by them.^6^

Evidence suggests that providing care for someone with palliative care needs can have a significant impact on the carer. This burden of caregiving has been shown to impact on a carers physical and psychological health and wellbeing, social relationships and connections,^7,8^ financial situation,^9,10^ working practices and employment.^11^

A 2019 report from Carers UK suggests around one in seven workers in the UK juggle paid work while caring for an older, disabled or ill person.^12^ Similarly high numbers of working carers have been reported internationally (e.g. Bijnsdorp et al.^13^), and many carers will have to give up paid employment altogether in order to provide care. For carers who remain in paid work, changes such as reducing working hours or moving jobs (often to lower paid jobs) may be required to accommodate the demands of caregiving. Even after a caring episode has ended, returning to work can be difficult and many carers never return to the labour market.^11^

Although the majority of this evidence comes from the wider carer literature and is not specific to carers of people with palliative care needs, the challenges are equally relevant for these family caregivers. Indeed, some of relrelevant for these family caregivers. Indeed, some of these issues are intensified for palliative care carers who are faced with a tumultuous, emotionally charged and often relatively short-lived episode of caregiving. This is combined with a sense of urgency, with palliative care carers reporting an obligation to ‘do anything it takes’ to support a person who has limited time left.^10^ Whilst evidence specific to carers of people with palliative care needs is limited, they have reported giving up work, reducing hours of work, or using up annual or sick leave to cope with the demands of caregiving.^9,13^ In addition, more than half of carers have been reported as making one or more employment transition over the palliative trajectory.^14^ These intersecting challenges lead to a gradual cumulative effect that weaken a carers employment prospects and ultimately their attachment to the labour market.

## Methods

### Research question

The aim of this study was to explore the perspectives and experiences of combining paid employment with palliative care family caregiving, and to assess the availability and suitability of employment support across three countries – the United Kingdom (UK), Aotearoa New Zealand and Canada.

### Design

A qualitative descriptive study design was used.^15^ Qualitative descriptive approaches draw from the tenets of naturalistic enquiry and are particularly relevant for capturing comprehensive perspectives of a phenomena.

### Setting and sample

We aimed to recruit participants from six countries with similarly performing health care systems and where English was widely spoken (UK, Aotearoa New Zealand, Canada, USA, Ireland and Australia). We selected countries with a similar level of health system development, palliative care development^16,17^ and similarities in welfare provision.^18^ Participants in this study comprised those with professional or personal experience in palliative care, who had participated in a previous survey study.^18^ This included health and social care professionals, charitable or advocacy workers, patients with palliative care needs and their family members, and bereaved family carers. For the purposes of this study family caregivers were defined as ‘carers, who may or may not be family members, who are lay people in a close supportive role who share in the illness experience and undertake vital care work and emotional management’.^19^

### Recruitment

Participants were recruited from 99 respondents who participated in a previous cross-sectional survey study, which identified and compared sources of financial support for palliative care family caregivers, across six countries (for full methods of the previous study see Gardiner et al.^18^). In this previous study we recruited patients with palliative care needs, family caregivers and professional experts in palliative care from each of the six countries. Participants completed an on-line survey on financial support available in their country, for palliative care family carers. At the end of the survey respondents were asked for their permission to be contacted about a subsequent interview study. Those who agreed to be contacted comprised the sample frame for the current study and were invited to participate via an e-mail containing information about the study.

We purposively sampled respondents with a view to achieving a maximum variation in experience sample, useful for understanding complex phenomena such as employment and caregiving.^20^ We aimed to achieve both demographic variation (gender, age, country) and phenomenal variation (whether a patient, carer or expert) to understand the phenomena from multiple perspectives. This is particularly useful when dealing with a subject that is closely constrained by policy, where individual experiences are informative, but an understanding of what is achievable within a given policy context is also required.^20^

Sample size was driven by an ‘Information Power’ approach, which takes account of a broad set of methodological considerations including study aim, sample specificity, theoretical background, quality of dialogue, and strategy for analysis.^21,22^ Sample size was not decided a priori but was established iteratively, based on the quality of data and sample characteristics. Sample size was also influenced by pragmatism, and unfortunately despite best efforts no participants could be recruited from three of the six countries involved (Ireland, USA, Australia).

### Data collection

Those who agreed to participate were invited to take part in a semi-structured interview, using either telephone or Skype. Participants were advised to find a quiet and private location for interviews, and to the best of our knowledge no one else was present during any of the interviews. Interviews were undertaken in 2017/18 by CG, BT and HG who have all received training in qualitative methods. A question guide for the interviews was developed by consensus on the basis of our previous research^9,18^ and is provided in Table 1. Interviews sought views and experiences on the financial costs of family caregiving in palliative care generally, with a specific focus on employment and the challenges of combining caring with paid work. Interviews were digitally recorded and field notes were taken where appropriate, interviews lasted between 20 and 60 min (mean 44 min)

**Table 1.** Question prompts for qualitative interviews.

• Do you think the financial burden of caregiving for someone with palliative care needs/at the end of life is a significant issue in your country? • How well do you think financial support for caregivers has been implemented in your country? • What do you think are the main barriers to uptake of financial support for caregivers in your country? *[prompts: eligibility restrictions around employment]* • Are their particular groups of carers who are at risk of not accessing support? *[prompts: in paid employment; retired; self-employed]* • What sort of things could improve uptake of financial support for caregivers in your country? *[prompts: better information provision; easier application process]* • Is there a role for health and social care professionals in the provision of information regarding financial support? • How do you think carers in your country could be better supported with regard to the financial burden of caregiving? • Do you know of current plans in policy or service development to address financial burden of caregiving? Or ongoing research programmes to explore it? *[prompts: changes to employment law]* • What policy chances, if any, are needed to improve financial support for caregivers in your country? *[prompts: welfare and benefit schemes, employment entitlements, carers allowances etc]* • What do you think are the main priorities for future research in this area?

### Data analysis

Interviews were transcribed verbatim by a professional transcription service, transcripts were not returned to participants for checking. Transcripts were read and fully coded by two of the authors (C.G. and B.T), and core thematic categories were identified by tagging groups of words with similar meanings. An initial coding framework was developed by consensus. This involved systematically coding interesting features of the transcripts across the data set, and collating data relevant to each code. Codes were collated into potential themes, with each theme incorporating all data relevant to it. Themes were then checked against both the coded extracts and the entire data set.^23^ The software programme QSR NVivo12 was used to aid analysis. This paper reports themes relating to employment, subsequent publications will focus on other key themes.

### Ethical issues

Verbal consent was given at the beginning of each interview and ethical approval was granted by the Universities of Sheffield and Auckland.

### Patient and Public Involvement (PPI)

Patient and Public Involvement (PPI) was provided by the Sheffield Palliative Care Studies Advisory Group, who are a lay panel working with the University of Sheffield. The study was presented to them at the protocol development stage and their feedback was incorporated into the final proposal. This included, for example, modifying the sampling strategy to include both patients and carers, rather than carers alone.

## Results

In total 53 survey respondents were contacted to invite them to participate in an interview, of these 30 agreed to participate and were interviewed. Fifteen were from the UK, nine from Canada and six from Aotearoa New Zealand. No participants could be recruited from either Ireland, USA or Australia despite e-mail invitations and reminders. See Table 2 for demographic details of participants.

**Table 2.** Demographics details of participants (*n* = 30).

	Total % (*n*)	UK (%)	Canada (%)	New Zealand (%)
Participants	30 (100)	15 (50)	9 (30)	6 (20)
Gender (female)	27 (90)	13 (87)	8 (89)	6 (100)
Role				
Patient	1 (3)	1 (7)	0 (0)	0 (0)
Carer/bereaved carer	10 (33)	7 (47)	3 (33)	0 (0)
Health care professional	11 (37)	4 (27)	3 (33)	4 (67)
Social worker	5 (17)	0 (0)	3 (33)	2 (33)
Advocacy/charity worker	3 (10)	3 (20)	0 (0)	0 (0)

Four main themes were identified from the analysis: changes to working practices for carers of people with palliative care needs; implications of combining caregiving and employment; employer support for working palliative care caregivers; and national, federal and government benefits for working carers. Themes are described in detail below.

### Changes to working practices for carers of people with palliative care needs

Some participants described changes to carers working practices necessitated by the demands of caregiving. These included reducing working hours, changing working patterns, changing jobs or giving up work altogether (either temporarily or permanently). Having to give up employment entirely could erode a person’s sense of self-worth and confidence, leading to a sense of dependency on welfare and difficulties returning to the labour market.

*‘Then, to go back to work, after spending 3 years caring for Mam, full-time. You’ve lost your place in society, and in the work place. And unless you experience it, you wouldn’t understand- I would never have thought this. . . you’ve lost your confidence’. (Bereaved caregiver, UK)*

*‘You take two years out of work and then to go back into the workforce, is your job going to be open for you? There’s no support for people in that situation’. (Healthcare Professional, New Zealand)*

Self-employed carers faced a particularly challenging situation, where it was often impossible to reduce hours or change working practices, forcing some to give up work altogether.

*‘I was self-employed, and I had a bed and breakfast, but really, I did it for the first year with Mum, but it was just too difficult. So I lost my business really’ (Bereaved caregiver, UK)*

### Implications of combining caregiving and employment

Combining caregiving with employment could have significant practical and health related implications for both patient and carer. In some instances, a patient’s concerns over the impact of their illness on a family members employment could influence treatment decisions.

*‘It is something that the patients worry about definitely. There’s a definite concern, because they don’t want to put their son or daughters or partners’ job at risk, and I think that sort-of may be a blocker to treatment sometimes’ (Healthcare professional, UK)*

Some working family carers had to employ paid carers in order to be able to continue in full time employment, but would then be providing care themselves during evenings and weekends, with implications for their own health and wellbeing. In some instances, the need for a family carer to remain in employment meant the patient had to be admitted to a residential facility, hospice or hospital.

*‘Sometimes they’ve [carer] tried putting their personal support workers in you know like Monday to Friday 8-4. And even if they do come every day, when the person gets home it’s like ‘okay now it’s job number 2’ and the weekend they’re continually you know really working. . . erm. . . so they get really get worn down pretty quickly. Erm so really what ends up happening, especially if someone’s still working – is they [cared for person] come into hospital so that’s the only option for them so they can work during the day’. (Healthcare professional, Canada)*

Participants spoke of carers being ‘pulled between’ the need to remain in employment to guarantee an income, and providing for the needs of the cared for person. Employment related challenges were unsurprisingly most pressing for people under retirement age, where an inability to work resulted in the greatest disruption to income.

*‘and the issues tend to come when we see patients who are much younger. . . and whose carers are possibly working, or trying to work, and juggle the appointments, and. . . taking time off work to attend appointments’. (Healthcare professional, UK)*

### Employer support for working carers of people with palliative care needs

Support from an employer was key to carers being able to juggle caring and work. Support from employers came in the form of either informal support (based on employer goodwill) or formal support (written into terms of employment). Employers who were perceived as supportive were generous with leave entitlements, flexible working to accommodate caring responsibilities, and bereavement leave. Often these arrangements were agreed outside of contractual obligations, and were made on the basis of a goodwill agreement. Carers were aware of the fragility of these informal arrangements, which were entirely dependent on the generosity of their employer, rather than a legal right.

*‘I think it really depends on . . . I think because my employer’s healthcare related, I think they are more considerate, I think if you were in another industry it’d be a very hard path. I think you might have to have quit your work, in order to have time off’ (Bereaved caregiver, Canada)*

*‘Erm, my employer was very generous. Erm and was really err, they were accommodating, (mmm) like really accommodating, err to me and my family. I’d had a long history [with the company] perhaps some of the rules were err, um, interpreted for me?’ (Bereaved caregiver, Canada)*

Some employers offered formal support for working caregivers, written into terms and conditions of employment and including flexible working, temporary reduction in hours or compassionate leave. In general, larger employers were most likely to offer comprehensive formal support packages, and highly educated employees in professional or higher paid roles were most likely to benefit. Smaller businesses and organisations with few staff could find it particularly hard to offer a full package of support to working caregivers.

*‘I think it’s like everything . . . you’ve got to sort-of find some balance, and how much it’s going to cost sort-of smaller companies compared to your bigger companies. I think that’s a hard thing to sort-of. . . I mean we hear about maternity leave changes, things like that, or . . . it’s the smaller companies that struggle the most with that’ (Healthcare professional, New Zealand)*

Some participants described how working carers re-purposed other employment entitlements to support caring, for example being ‘signed off’ by their GP so they could use sick leave to provide care. Others used up annual leave to provide care.

*‘No, no he didn’t. He wasn’t given any time off, he just took his holidays to look after me’. (Patient, UK)*

### National, federal and government benefits for working carers

Government or state benefits were sometimes used by working carers, for example the UK Carer’s Allowance, Aotearoa New Zealand Supported Living Payment or Canadian Compassionate Care Benefit. However, it was noted that these benefits are not comprehensive, for example the Aotearoa New Zealand Supported Living Payment is not available for those caring for a partner/spouse. In addition, some of these benefits come with restrictions on employment (e.g. to be eligible for the UK Carer’s Allowance in 2021 you must earn less than £128 a week), effectively forcing carers to choose between caring or substantive employment. Other government policies designed to support working carers were perceived by some, not to be fit for purpose. For example, whilst UK employees have a legal right to take time off work to provide care, there is no right to be paid during any leave taken, raising questions for participants over who might benefit.

*‘And there’s no automatic right to be paid [during care leave], so I would wonder if any carers at all take that up?’ (Healthcare professional, UK)*

Some carers had private insurance that allowed them to take time off work to care, but this was usually those in higher paid or professional jobs.

*‘And, erm, I had, probably about a month and a bit of short term disability [paid sick leave]. And then I ended up getting, I think, through my employment insurance, erm, all of this is a blur (yeah I’m sure it is), but I remember getting some . . . and then I did apply for long term disability’ (Bereaved carer, Canada)*

The Canadian Compassionate Care Benefit (CCB) is the only government funded benefit specifically aimed at working palliative care carers. This provides up to 55% of earnings for up to 6 months, for carers of people in the last 6 months of life. In general, the CCB was perceived as a useful benefit with high uptake, which supported working carers to remain in employment. Nonetheless some challenges remained, most apparent of which was the maximum limit of 55% of earnings which for some carers (particularly those on lower incomes) was insufficient to live on.

*‘I think if you’re able to take the time off it’s great but a lot of people aren’t because they still need to have their full income’. (Healthcare professional, Canada)*

*‘But if you’re already starting, y’know just over the minimum wage, or, y’know you’re not making very much money. To then cut that to 55%, I mean people then aren’t able to take compassionate care [benefit], because then they’re not able to pay rent, or make some mortgage payment or something like that, right?’ (Healthcare professional, Canada)*

A further issue was deciding when to take the CCB, as often it was not possible to predict with any accuracy when someone was within 6 months of death. This was a particular problem for carers of patients with non-cancer conditions which often have less predictable disease trajectories.

*‘Motor Neurone Disease is a difficult one (umm) for people to know when to take the compassionate care leave’. (Healthcare professional, Canada)*

## Discussion

### Main finding

This study reports some of the key challenges facing working family carers caring for someone with palliative care needs across the UK, Aotearoa New Zealand and Canada. Working carers often have to adapt and modify their paid working practices in order to combine employment and caregiving. Our data suggests that juggling family caregiving with paid employment has significant negative implications for both patient and caregiver, a concerning finding given the reliance within Western healthcare systems on family carers.^1–3^ Nonetheless, caregivers can be supported to remain in employment and we identified various ways that both employers and governments can help carers to remain in the workforce.

Across the three countries we included, various examples of government legislation and policy were described, designed to support working carers. However, there was considerable variation in the perceived benefit of such legislation. International evidence suggests that employment legislation can be effective in mitigating the negative impacts of caring on employment (e.g. Carers UK^12^). However, as we reported in our findings such legislation is often inconsistent, variable and open to a considerable degree of interpretation by employers. For example, whilst family carers in the UK and New Zealand have a legal right to request flexible working to provide care, there is no incentive for employers to accept a request, nor any obligation to provide payment.^24–26^ Our findings indicate that the ambiguity of such policies is likely to limit who is able to benefit.

Employment legislation alone is unlikely to provide sufficient support for working palliative care carers. Our findings suggest that short-term government benefits and welfare packages are also crucial for supporting working carers. Canada is the only country in this study which offers a specific benefit (Compassionate Care Benefit) for working palliative care caregivers.^26^ Evidence suggests the CCB has the potential to serve as an important public health intervention to address the needs of palliative care caregivers^27^ and may serve as a model for other countries. However, whilst the introduction of the CCB has been generally well received, concerns have been raised over equity, with evidence suggesting women and the socio-economically disadvantaged are often excluded from the programme.^28^ Our findings, whilst indicating the CCB was well used, also highlighted some limitations particularly for those carers on a low income. Whilst such schemes are not without their challenges, the implementation of formal paid care leave is an obvious recommendation for those countries with no such arrangements currently in place. The cost of these schemes is likely to be partially or fully offset by economic gains in worker retention, and reductions in absenteeism and paid leave (e.g. Carers UK^12^). Further research is required to assess the feasibility of transferring schemes such as the Canadian CCB to other countries, and to explore ways to improve equity in employment support.

Whilst government and state support is crucial, our findings also suggest an important role for employers in supporting working carers, with both formal and informal support valued. This finding is supported by evidence from a recent survey of unpaid carers in the UK who reported a supportive employer/line manager and flexible working arrangements were the most important factors influencing employment retention.^12^ However our study found that, in reality, whether or not someone received workplace support was often based on the goodwill of employers, or was limited to highly educated employees in higher paid, professional roles. This issue of equity has been highlighted by others with Hulme et al.^11^ arguing that employment status may represent an equity issue for palliative care carers, noting for example that carers earn less than non-carers.^11,29^ In order to improve the consistency and equity of employment support, incentives to adopt supportive workplace policies need to be provided, and the benefits of supporting working carers highlighted. For example, it has been estimated that UK businesses could save up to £4.8 billion a year in unplanned absences and a further £3.4 billion in improved employee retention by adopting flexible working policies to support those with caring responsibilities.^12^

Political and economic landscapes also play a role in influencing employment challenges related to caregiving, most notably planned increases in statutory retirement/pension age. In the UK and New Zealand retirement/state pension age will rise to 67 by 2028 and 2040 respectively, and further subsequent rises are expected.^30,31^ Changes such as these have profound implications for palliative care caregiving. Our evidence suggests that carers under retirement age face disproportionate challenges, yet in coming years more people will begin a caregiving episode whilst in paid employment, and employed caregivers will be forced to remain in the labour market for longer. The economic and personal consequences of these changes will be significant, reinforcing the need for immediate action.

### Strengths and limitations

This study of three countries has international relevance. However, few participants were recruited from Aotearoa New Zealand and patients and charity workers were only recruited in the UK sample; this limits generalisability across all countries. Differences between the three countries in welfare provision means international comparisons should be treated with caution. Data were collected in 2017–2018 and more recent data may reveal different findings, especially in light of the COVID-19 pandemic and the impact on both employment and caregiving.

## Conclusion

Working palliative care caregivers face a range of challenges which impact on their ability to continue caregiving and their likelihood of remaining in, or returning to, employment. Evidence from the UK, Aotearoa New Zealand and Canada highlights a range of issues faced by working carers, with marked similarities across all three countries. Supporting carers to remain in work has potential benefits for the person with palliative care needs, the caregiver, and the wider economy and labour market. However, working family caregivers need greater support to be able to remain in employment. Further research is required to confirm our findings, particularly in Aotearoa New Zealand where only six participants were recruited. Nonetheless employers, policymakers and governments have a role to play in developing and implementing policies to support working carers to remain in employment.
